# Regulated timber harvesting does not reduce koala density in north-east forests of New South Wales

**DOI:** 10.1038/s41598-022-08013-6

**Published:** 2022-03-10

**Authors:** Brad Law, Leroy Gonsalves, Joanna Burgar, Traecey Brassil, Isobel Kerr, Chris O’Loughlin, Phil Eichinski, Paul Roe

**Affiliations:** 1 Forest Science, NSW Primary Industries, Parramatta, Australia; 2 Vancouver, BC Canada; 3 Queensland University of Technology, Brisbane, Australia

**Keywords:** Ecology, Ecology, Environmental sciences

## Abstract

The compatibility of forestry and koala conservation is a controversial issue. We used a BACIPS design to assess change in koala density after selective harvesting with regulations to protect environmental values. We also assessed additional sites heavily harvested 5–10 years previously, now dominated by young regeneration. We used replicate arrays of acoustic sensors and spatial count modelling of male bellowing to estimate male koala density over 3600 ha. Paired sites in nearby National Parks served as controls. Naïve occupancy was close to 100% before and after harvesting, indicating koalas were widespread across all arrays. Average density was higher than expected for forests in NSW, varying between arrays from 0.03–0.08 males ha^−1^. There was no significant effect of selective harvesting on density and little change evident between years. Density 5–10 years after previous heavy harvesting was equivalent to controls, with one harvested array supporting the second highest density in the study. Within arrays, density was similar between areas mapped as selectively harvested or excluded from harvest. Density was also high in young regeneration 5–10 years after heavy harvesting. We conclude that native forestry regulations provided sufficient habitat for koalas to maintain their density, both immediately after selective harvesting and 5–10 years after heavy harvesting.

## Introduction

Changes in land-use for human production affect many species and protecting biodiversity in these areas is a key challenge for achieving ecological sustainability. In particular, threatened species often have specialised requirements that need to be considered when mitigating impacts of changed land-use. Mitigations can be encoded in regulations, such as in forestry practices, but the effectiveness of these needs to be assessed to ensure adaptive management and ongoing improvement^1,2^. Example mitigations that can benefit a range of species include retention level during harvesting and the extent of old forest^3,4^.

The koala *Phascolarctos cinereus* is an iconic arboreal marsupial that is declining in a significant part of its range, where it is listed as an endangered species^5^. Although mobile across highly modified landscapes^6,7^, koalas are impacted by permanent tree cover loss and fragmentation as well as increased urban development around bushland, road traffic, dog attack, climate change and disease (e.g.^5,8,9^). Forestry is one land-use that overlaps extensively with koala habitat.

The influence of forestry on koalas is controversial, although current evidence suggests regulated harvesting with environmental protections could be compatible with koala conservation. For example, a radio-tracking study in the Pilliga forests of New South Wales (NSW) found that koalas tolerate selective harvesting of shelter trees, at least in the short term (i.e. 6 months after harvesting)^10^. In tall hinterland forests of north-east NSW, a regional survey mostly recorded koalas in regrowth forest (< 30 years old), though the rate of detection was low and confounded with low elevation^11^. Koala scats and vegetation in their home range are also correlated positively with the number of selective harvesting events, suggesting koala populations are resilient to historical harvesting^12–14^. But scats are also associated with structurally complex, uneven-aged forests with some mature and old-growth elements, a large basal area and mixed species associations dominated by preferred browse species^13^. Such surveys are limited by not accounting for imperfect detection and so results must be interpreted cautiously. The most comprehensive survey that did account for imperfect detection used passive acoustics to target forests with different timber harvest treatments and found that occupancy and bellow rate were not related to harvest intensity, time since harvest or extent of old growth^15^. Occupancy based on acoustic sampling is an effective response variable for koalas because their density is typically low^13,16^ and only one koala (male) would be expected to occupy the range detected by each sensor (~ 30 ha for koalas; Law unpubl. data).

Nonetheless, changes in density provide a more detailed picture of the effects of different land use on species, albeit at a more localised scale, than occupancy metrics. Recent studies have shown koala density can be high in areas dominated by young trees if they are a preferred browse species. For example, young blue gum *Eucalyptus globulus* plantations in Victoria were found to support higher mean koala density (0.85 ha^−1^) than either native vegetation blocks (0.68 ha^−1^) or native strips (0.66 ha^−1^)^17^. Elsewhere, koalas use areas dominated by regenerating trees on mine sites^18^. A remaining knowledge gap concerning koalas and forestry is the extent to which density is impacted by harvesting, especially for native forests where browse trees are removed, but allowed to regenerate naturally under regulations designed to protect environmental values.

Our study aimed to assess the effects of regulated timber harvesting on koala density in public native forests of north-east NSW. We used a before-after-control-impact-paired-series (BACIPS) experiment to assess change in density. Because forests of north-east NSW are naturally heterogenous and on average 40% of timber production forests are excluded from harvesting via regulations to protect environmental values^1^, we assessed male koala density over 400 ha study areas using arrays of acoustic sensors to incorporate harvesting exclusions and spatial count (SC) modelling to estimate density^19^. Acoustic arrays and SC modelling generally produce plausible and reliable estimates of koala density in NSW^20^. We predicted density to be lower in net harvested areas, but similar or greater (if home ranges shifted) in surrounding harvest exclusions. To provide further context to the immediate effects of timber harvesting, we also sampled additional sites that were heavily harvested 5–10 years previously. Such sites comprised a mosaic of regenerating eucalypts in previously harvested areas interspersed with mature forest in harvesting exclusion zones. We predicted koala density would have partly recovered and be approaching that estimated in control sites within nearby National Parks.

## Results

### Harvesting

Selective harvesting at three BACIPS treatment sites in 2020 resulted in a gradient of harvest intensity with timber volumes removed varying from 17–51 m^3^ ha^−1^ (Table 1). Harvest intensity was greater in areas ‘heavily harvested’ 5–10 year previously, though it was variable between sites being substantially greater at Comboyne State Forest (Table 1).

**Table 1:** Harvesting details for three selectively harvested (BACI) sites and three previously heavily harvested sites. Note that mapping in the older harvests (pre-GPS in machinery) overestimated area harvested and therefore underestimated harvest intensity. Multiple years of harvest refer to harvesting in adjacent areas, not repeat harvesting.

State forest (compartments)	Year of harvest	Area of harvest (ha)	Total volume removed (m^3^)	Harvest intensity (m^3^/ha)
Cowarra	2020	264	6177	23
Kalateenee	2020	289	4771	17
Lower Bucca	2020	304	15,480	51
Comboyne (147, 148, 150, 151, 152)	2009, 2011, 2016	296	61,209	207
Cairncross (18, 19, 20, 21)	2012, 2013	466	18,370	39
Kiwarrak (52, 56, 57)	2011	169	9093	54

### Acoustic surveys for koalas

Acoustic sensors recorded for about 21,000 h in each of 2019 and 2020. The total number of validated koala bellows across our before-after arrays was 3958 in 2019 and 2552 in 2020 (six of 150 units failed in 2020 due to issues with clock batteries) as well as 2083 at three previously harvested arrays. Calling activity varied sixfold among arrays in 2019, from an average of at least one call in 8.9 ± 1.3 10-min periods per night at Kiwarrak SF down to at least one call in 1.4 ± 0.3 10-min periods per night at Cowarra SF. Similar variation was found in 2020.

Detection rates for koalas were very high across all sites before and after harvesting (Table 2). As part of the main experiment, koalas were detected at 97% of the 153 pre-harvest sensors set in 2019. In 2020, detection rates (i.e., naïve occupancy^21^) decreased slightly to 92% of the sensors set immediately post-harvest and 89% of the sensors set at control sites in National Parks. A 100% detection rate was recorded at the 77 sensors set at previously heavy harvested sites.

**Table 2:** Number of acoustic sensors per array and koala detection rates (naïve occupancy) at each site.

Array	# Sensors	% Koalas pre-harvest (2019)	% Koalas post-harvest (2020)	% Koalas 5–10 years post-harvest
Ulidarra NP (control)	25	100	96	–
Lower Bucca SF (treatment)	26	100	92	–
Bago Bluff NP (control)	25	96	71	–
Cowarra SF (treatment)	26	92	85	–
Kumbatine NP (control)	25	100	100	–
Kalateenee SF (treatment)	25	96	100	–
**5–10 years post-harvest**				
Kiwarrak SF	26	–	–	100
Comboyne SF	26	–	–	100
Cairncross SF	25	–	–	100
Total/mean	178	97	89	100

### Koala density estimates and the effect of harvesting

**Figure 1:**
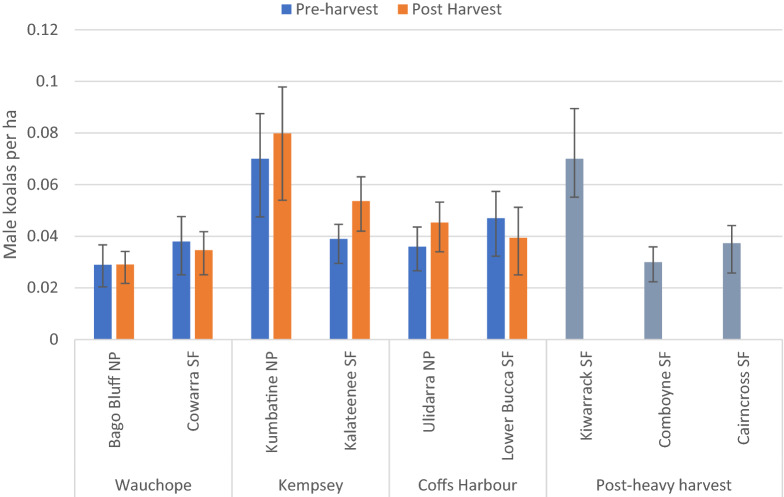
Male koala density before and after harvesting. Modelled male koala density (mean ± 50% credible interval) in pre- and post-harvest years at BACIP sites and three additional sites 5–10 years post-heavy harvest. Density was estimated by Spatial Count analysis of acoustic data collected from arrays.

Spatial count analyses estimated that average male koala density was higher than expected for hinterland forests in north-east NSW, varying between arrays from 0.03–0.08 ha^−1^ (strongly informative prior) (Fig. 1). There was negligible difference in density between paired arrays in State Forest and National Park. Kumbatine NP and Kiwarrak SF (non-paired) were notable for their higher densities. Credible intervals were wide for the mean estimates of density for most arrays. This is not unexpected given that arrays encompassed about 400 ha, including variable habitat suitability for koalas that was “averaged” across the array (see below).

**Figure 2:**
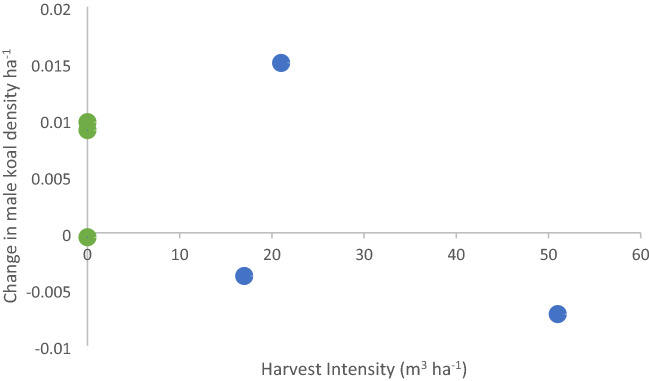
Change in male koala density in relation to selective harvest intensity. Change in density at control and harvest arrays in relation to harvest intensity measured as timber removed per hectare of harvest. Positive change indicates an increase in male koala density at the array, negative change indicates a decrease post-harvest. Green points are Control arrays and blue points are Harvested arrays.

There was also little change in koala density from pre to post harvest periods in either control National Parks or harvested State Forests, though there was a slight decrease at Cowarra and Lower Bucca and slight increase at Ulidarra, Kalateenee and Kumbatine. Statistical analysis following the BACIPS design confirmed there was no significant effect of selective harvesting on male koala density (mean difference_control-harvested_ = 0.0099 male koalas ha^−1^; paired t = − 2.67, p = 0.12). Despite no overall difference in density, Lower Bucca recorded the highest harvest intensity (51 m^3^ ha^−1^; standing timber volume removed) and the largest decrease in male koalas ha^−1^, albeit a relatively small change of − 0.007 males ha^−1^ (Fig. 2). Moreover, male density remained high 5–10 years after heavy harvesting (Fig. 1). Density estimates at three sites with older heavy harvesting were equivalent to controls, with one site (Kiwarrak SF) having the second highest density found in the study, although we acknowledge that pre-harvest density at these three sites was unknown.

### Spatial variation in koala density

**Figure 3:**
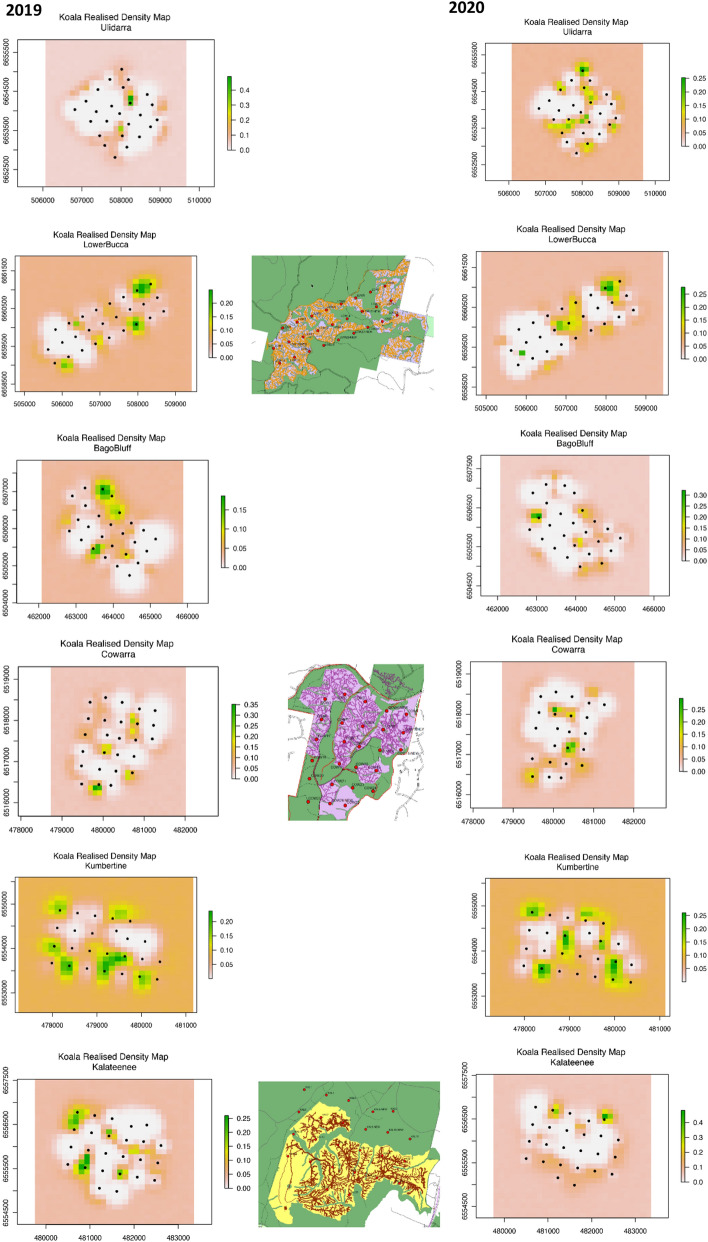
Spatial variation in male koala density across each array before (2019) and after (2020) harvesting with paired control sites in National Parks (BACIPS experiment). Legend is males ha^−1^, but note different scales for each array. Harvest area (coloured), harvest tracks and arrays (dots) are also shown for the three treatment areas. Maps were created using ArcGIS 10.4.1 for Desktop (https://esriaustralia.com.au/arcgis-desktop) from GPS track logs in harvester machines and koala density outputs from Spatial Count modelling using JAGS (ver 4.2.0), interfacing through R using the rjags package.

**Figure 4:**
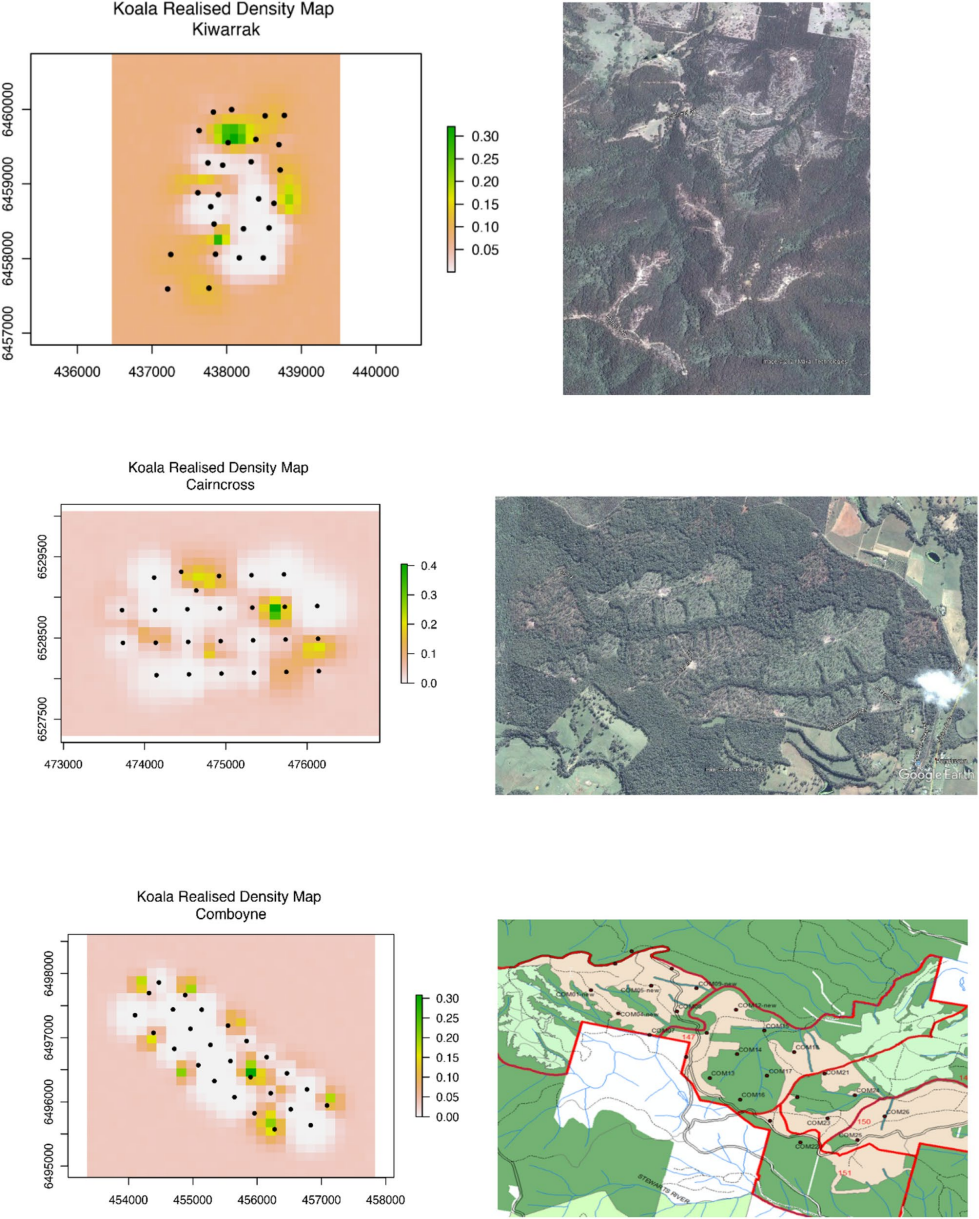
Spatial variation in male density 5–10 years after heavy harvesting. Area of harvest and retention (Google Earth) is presented for comparison as they appeared immediately after harvest. Because Comboyne was extensively harvested over a number of years composite harvests are shown as a map (more recent harvesting (2016–2018) also took place outside the grid in adjacent compartments (light green) prior to deployment of array in 2020). Black dots show sensor locations. Legend is males ha^−1^, but note different scales for each array. Maps were created using ArcGIS 10.4.1 for Desktop (https://esriaustralia.com.au/arcgis-desktop) from compartment harvest history data or Google Earth imagery and koala density outputs from Spatial Count modelling using JAGS (ver 4.2.0), interfacing through R using the rjags package.

Male koala density was highly variable within each array, and included areas equivalent to the “average” density for the array (0.03–0.07 males ha^−1^) as well as above (e.g. 0.3 males ha^−1^) and below average density (e.g. < 0.01 males ha^−1^) (Fig. 3). A common feature across all arrays was close to 100% detection at each sensor (as noted above), even where density was predicted to be low, as well as small hot spots of higher density. Hot spots were localised areas of above average density, with typically 2–4 hot spots per array. For example, Ulidarra NP in 2019 was characterised by a broad extent of lower density areas, punctuated with just two small hot spots of estimated higher density. In contrast, Lower Bucca SF and Kumbatine NP exhibited a broad extent of average density, also punctuated with five hot spots of higher density. Given the change from drought to a wet year and harvesting in State forest sites, it's not surprising that there was also some spatial variation in density between years, even in control sites, though this mainly reflected small shifts in activity centres. The influence of harvesting at this finer scale is apparent where hot spots of activity sometimes coincided with harvesting, while in other harvested areas density noticeably decreased between years. Similar variability in koala density within an array was observed 5–10 years after heavy harvesting (Fig. 4).

**Figure 5:**
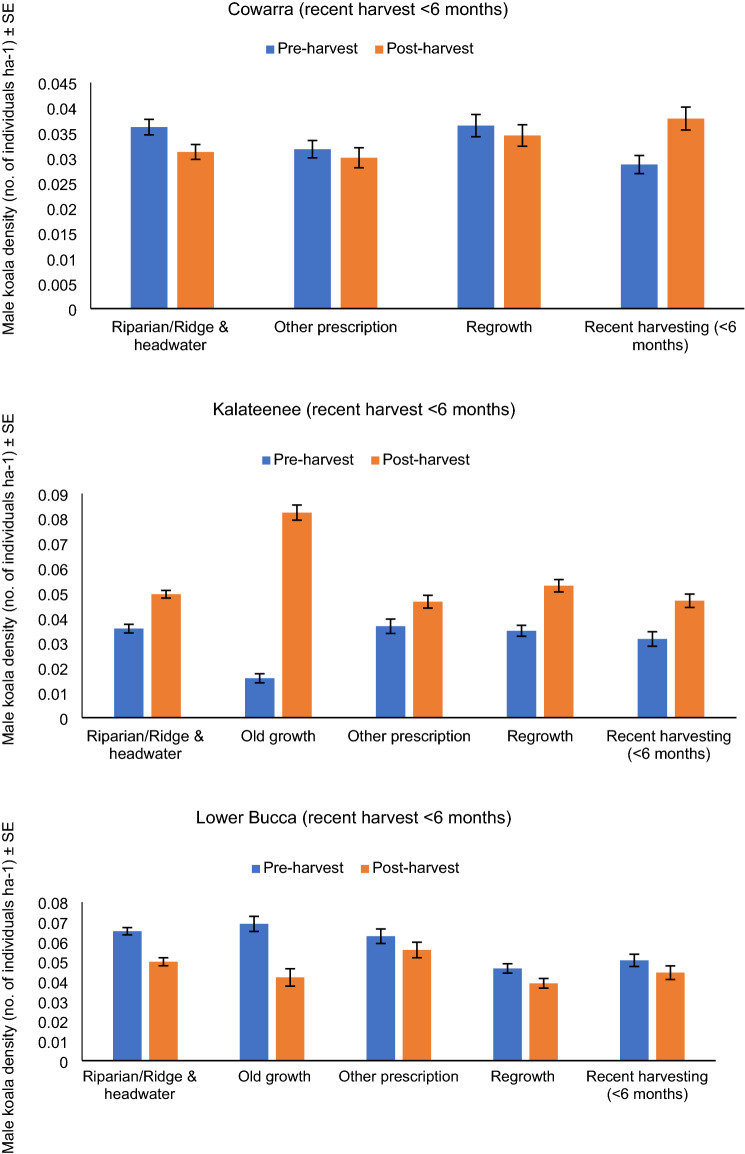
Male koala density in different forest age classes/harvesting prescriptions (exclusions). Mean male density before and after selectively harvesting at three acoustic arrays.

**Figure 6:**
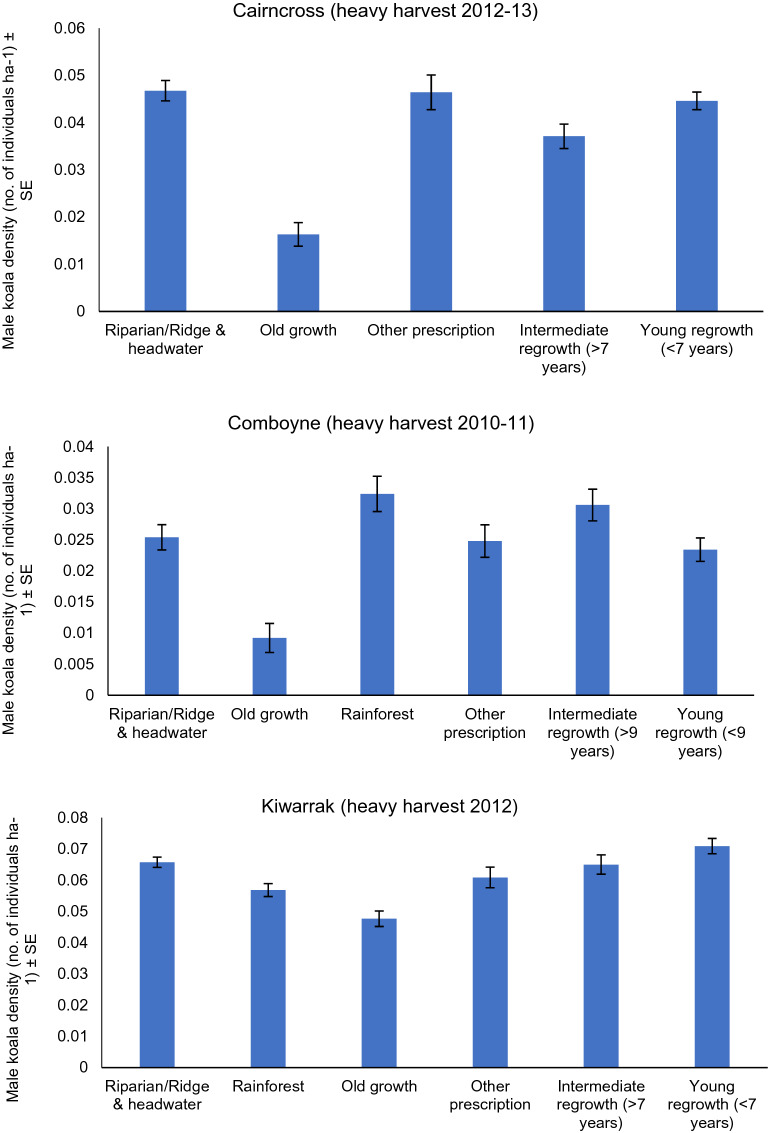
Male koala density in different forest age classes/harvesting prescriptions (exclusions). Mean male koala density in different forest age classes/harvesting prescriptions (exclusions) 5–10 years after heavy harvesting at each of three acoustic arrays.

When the spatial variation in density was overlayed at each site with different forest age classes, areas that were recently selectively harvested displayed only minor changes compared to pre-harvest density and density in these areas was generally comparable to density in areas classified as old growth, riparian-ridge headwaters, other prescriptions or older regrowth (Fig. 5). A similar pattern was evident at sites heavily harvested 5–10 years previously, except old growth consistently supported the lowest density of male koalas and areas of young regeneration (< 7–9 years) supported among the highest density in two of three areas (Fig. 6).

## Discussion

This is the first study to directly assess the effects of native forest harvesting on koala density. A carefully designed BACIPS experiment was undertaken with each study area spanning about 400 ha to encompass the locally heterogeneous landscape. While passive acoustic recording mainly detects male koalas, calling is a sign of reproductive activity by adults rather than juveniles, and it is also energetically expensive^22–24^. Moreover, sex ratio is typically thought to be 1:1 in koala populations^14,25–27^, and this was confirmed at one of our study areas using fresh scats and genotyping (Kalateenee State Forest; Gonsalves et al. unpubl. data). Acoustic results show male koalas were widespread in all our sites and that selective harvesting had no immediate effect (< 6 months) on their density. Moreover, koala density was also equivalent to controls 5–10 years after extensive, heavy harvesting and spatial variation in density at an array was not negatively related to those areas recently harvested. A density estimate at one site (Kalateenee SF) was also derived from genotype analysis of fresh scats and this was consistent with the estimate derived from the acoustic array (Gonsalves et al. unpubl. data). Acoustic arrays and SC modelling have previously been demonstrated to produce plausible estimates of male koala density^20^. Other methods can be used to estimate koala density, such as mark recapture or drones^28,29^, but they were considered logistically too difficult to implement in tall dense forest and/or too costly for the scale of the current project where density estimates were produced over > 3600 ha of forest with requirement for repeating after harvesting. One noteworthy difference in density of matched pairs was between Kalateenee State Forest and Kumbatine National Park, where the park supported almost twice the density as the adjacent state forest. This difference may be due, at least in part, to fire history as Kumbatine was last burnt by wildfire 15 years earlier compared to only 3 years at Kalateenee. Kiwarrak was our other high-density area and it had not previously experienced a wildfire for 41 years. A parallel study using acoustic arrays found that koala density declined dramatically after high severity fire^30^. We consider the implications of these findings in relation to harvesting in more detail below.

First, koalas have previously been shown to tolerate disturbance. In a radio-tracking study in the Pilliga, koalas continued to occupy their home ranges immediately after selective logging of shelter trees^10^. A recent study also found that koala survival was high after intensive harvesting of blue gum plantations in Victoria and that they responded to clearfall harvest by moving into adjacent habitat^31^. These studies are consistent with our findings and suggest that during and after selective harvests koalas will continue to occupy their home ranges. More intensive harvests may push koalas into adjacent exclusion areas, until the disturbance passes and/or suitable habitat regenerates. On average, about 40% of state forests in NSW State forests are excluded from harvesting for environmental reasons and there are also tree retention requirements within the harvested area, providing refuge habitat and connectivity^1^ (see also “Methods”).

Continued widespread detections at our sites and maintenance of moderate density 5–10 years after heavy harvesting suggests koalas can and do rapidly exploit the young regenerating forest after harvest, providing further support for the effectiveness of existing forestry regulations. Koalas also use regenerating forest on rehabilitated mining areas as young as 6 years post rehabilitation, where they bred with good body condition, spending equivalent time in rehabilitated and undisturbed areas^18^. Young blue gum *Eucalyptus globulus* plantations in Victoria also support higher mean koala density (0.85 ha^−1^) than either native vegetation blocks (0.68 ha^−1^) or native strips (0.66 ha^−1^)^17^, indicating koalas readily use habitat comprised of young preferred browse trees, even when in a monoculture. Indeed, GPS tracking of koalas in an early post-harvest landscape adjacent to our acoustic study areas found koalas use both young regenerating trees in the harvested area and mature forest within harvest exclusions (Law et al. unpubl. data). No detectable response of male koala density to timber harvesting also supports results at a metapopulation scale from regional occupancy surveys across 171 sites, in that neither occupancy nor bellowing activity were found to be related to time since harvest or harvest intensity^15^. It is notable that concurrent assessments of tree canopy cover found little change in species composition after harvesting, with > 14 tree species comprising the canopy^32^. The primary browse species for koalas, tallowwood *Eucalyptus microcorys*, represented a small portion of the canopy area at our sites and it was similar between control (2–3% cover) and harvested areas (5–6% cover), including those previously heavily harvested. *Eucalyptus microcorys* was the most widely distributed tree in the region, being recorded at 120 of 171 sites (4% cover)^15^, indicating resilience in a key tree species for koalas.

A powerful outcome of SC analyses is the ability to model spatial variation in density within each array and overlay this with different forest age classes or harvesting exclusions. Caution needs to be applied to avoid interpreting this modelling at overly fine resolutions, such as small patches of forest, given that koala bellows can be recorded at up to a 300 m radius. Nonetheless, this approach provides indicative comparisons of density at a finer resolution than the mean density estimated for the entire array (~ 400 ha), which typically encompasses a heterogeneous landscape.

Density overlayed on different forest categories within arrays found no support for lower koala density in those areas that were selectively harvested compared to other categories of forest. At sites 5–10 years after heavy harvesting, the areas dominated by young regenerating forest appeared to support higher koala density, suggesting koalas are regularly using such areas^17,18^. These findings are currently being further tested by GPS tracking of individual koalas and assessing home range overlap with different forest categories and use of trees of different sizes (Law et al. unpubl. data).

Avoiding mortality to koalas during harvest remains a challenge. Spotters have been used in blue gum plantations to reduce mortality^29^, but the cryptic nature of koalas suggests this would be unproductive in the tall coastal forests of NSW (authors pers. obs). Harvesting crews are, however, trained and required to look for koalas during forestry operations. Alternatively, temporary noise disturbance^33^ in advance of harvesting in native forests could be trialled to nudge koalas away from advancing machinery.

Management recommendations from this study support retention forestry as an effective approach to provide a mosaic of age classes and to mitigate habitat loss, especially immediately after harvest, and allow time for recovery. On average, 40–50% of state forests in NSW are excluded from harvesting^1^, but this retention has increased under newer regulations. Retention forestry is widely recommended as the best approach to achieve biodiversity conservation in timber production forests^34,35^, where it involves a network of exclusion zones as well as targeted retention within the net harvest area^1^. For koalas, targeted retention focused on browse trees needs to be fine-tuned using preference data for tree species, size, spatial configuration and the nutritional value of the different species. Triggered retention of browse species is now achieved through use of habitat suitability maps^36^ as per the newly established practice in NSW. Dispersal of harvesting in space and time within management zones also aids persistence over time while habitat recovers. Monitoring of koalas is essential to ensure management practices are achieving their aims of protecting koalas, to describe changes as the forests regenerate after harvesting and to inform the wider community of changes to the species status across multiple land tenures. Annual monitoring of koala occupancy has taken place since 2015 (https://www.dpi.nsw.gov.au/forestry/science/forest-ecology/koala-research-in-nsw-forests). Monitoring is also relevant in relation to devastating wildfires, which are a significant threat to koalas in forests^30,37^, as is the case for other threatened species^38,39^.

## Methods

### Study area and experimental design

The study was undertaken in hinterland forests of the mid-north coast of NSW, with treatment/control pairs located near Wauchope, Kempsey and Coffs Harbour. We followed a replicated BACIPS (Before–After-Control-Impact Paired Series) design, with paired treatment and control sites in State forest and nearby National Park (where there has been no recent harvesting), respectively. Pre-harvest density was sampled in the spring of 2019. Timber harvesting took place across the three treatment sites between June and October 2020 and we re-sampled 2–5 months after harvesting in spring 2020. We supplemented this design with acoustic arrays at three additional sites that had been more heavily harvested 5–10 years previously. These were located near Taree and Wauchope. All harvested State forest sites were dominated by regrowth forest that had been previously harvested over multiple rotations during the past 50–100 years.

**Figure 7:**
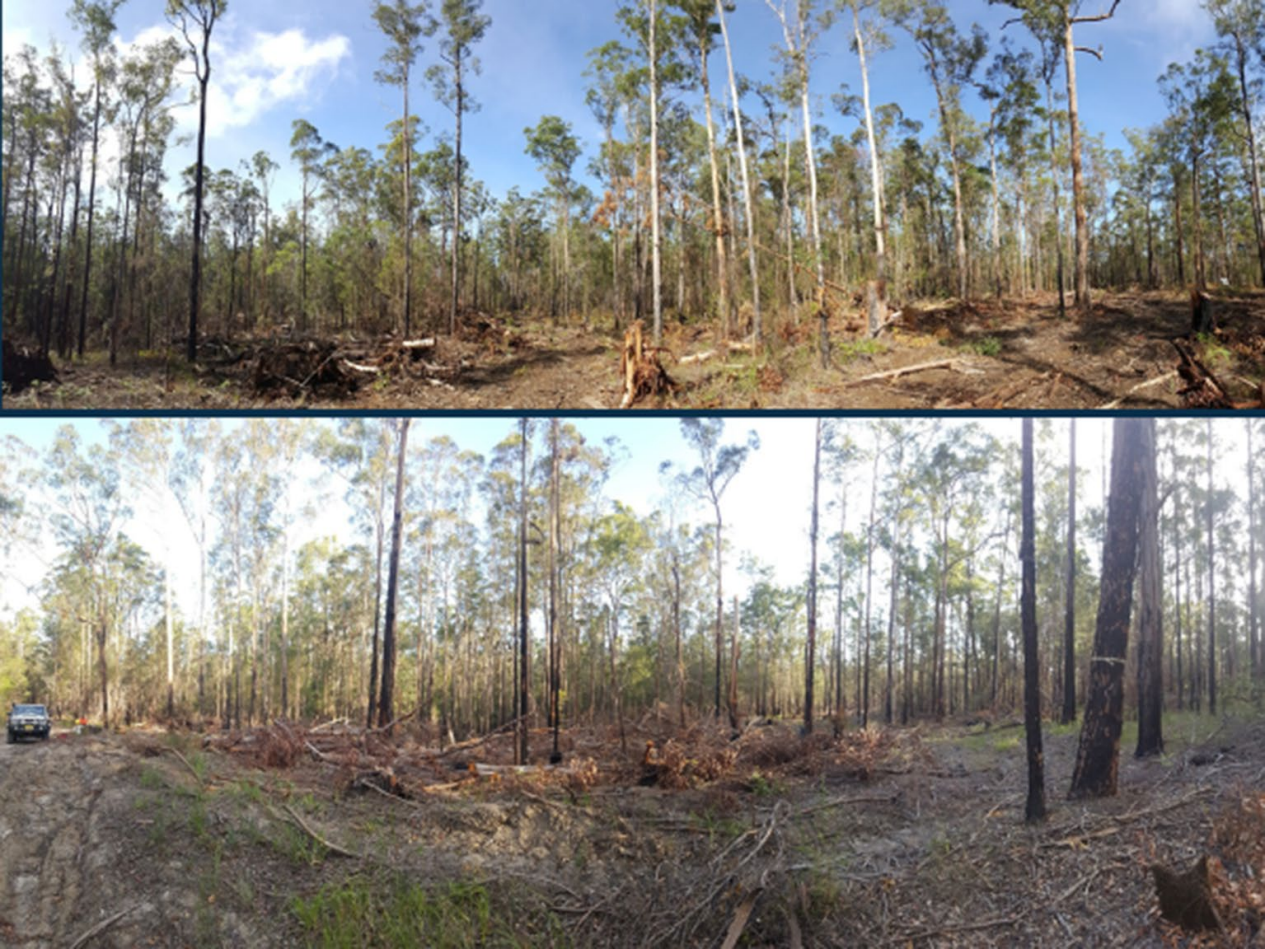
Selective harvesting and tree retention at Kalateenee State forest in 2020.

**Figure 8:**
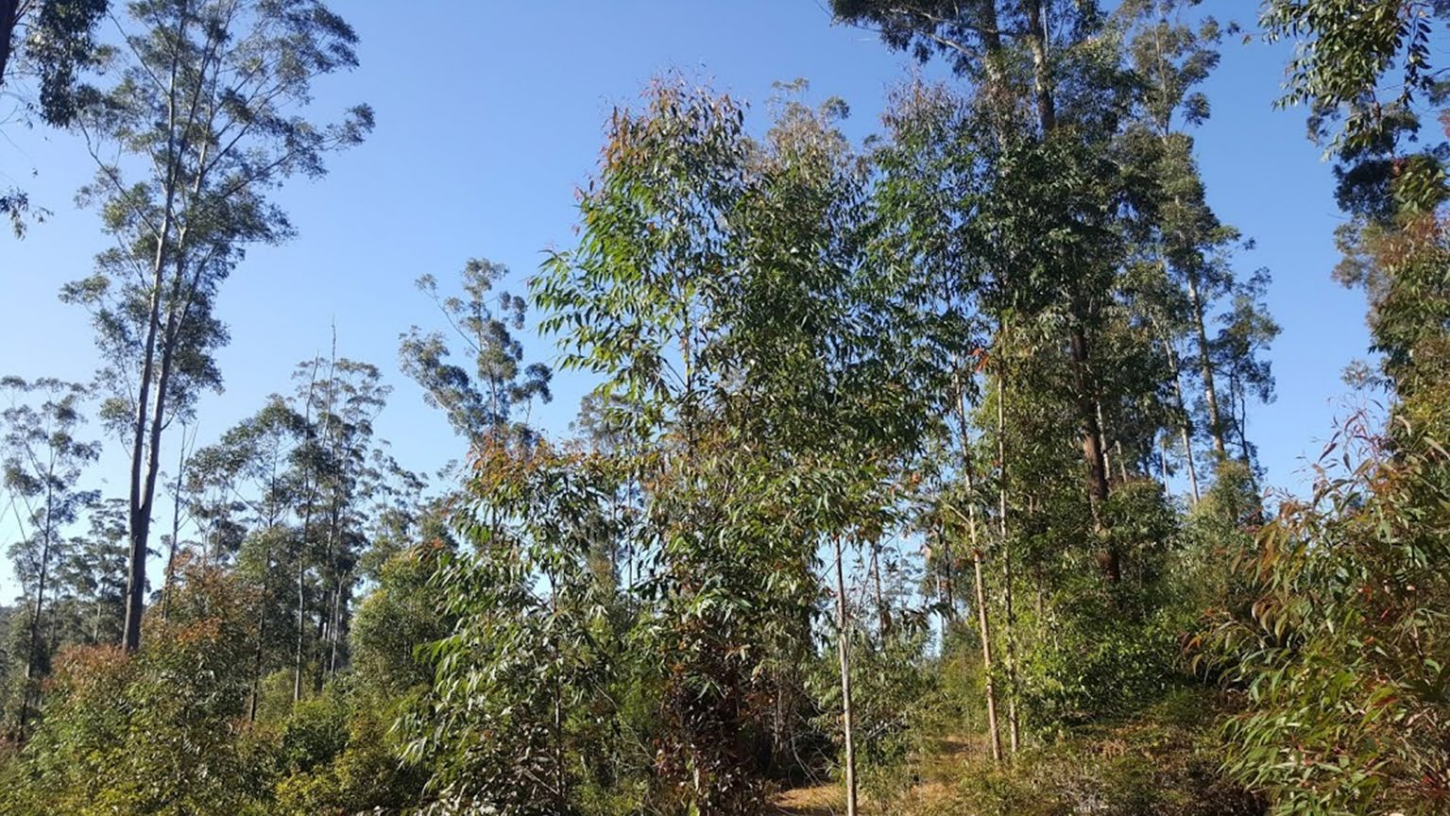
Young regeneration and blackbutt seed tree retention after heavy harvesting at Comboyne State Forest in 2016–2018.

Each forest comprised a mix of forest types, though blackbutt *Eucalyptus pilularis* was often a dominant, and tallowwood *E. microcorys* and grey gum *E. propinqua* were sub-dominants. Wetter forest types comprising Sydney blue gum *E. saligna*, flooded gum *E. grandis* and rainforest often dominated the gullies. A range of tree species were targeted for timber harvesting, though blackbutt was a preferred species. Many of these tree species are also browsed by koalas, with tallowwood a primary browse species. Harvest intensity at recent harvest sites was best described as selective (Fig. 7), where harvesting is inherently variable, but averaged retained basal area within a coupe being ≥ 10 m^2^ ha^−1^ and timber volumes removed < 55 m^3^ ha^−1^ (Table 1). The three supplementary sites were heavily harvested under regeneration silviculture as practiced at that time (Fig. 8). Standard mandatory environmental protections were employed, which in north-east NSW resulted in 40–50% of state forests being excluded from harvesting^1^. The first tier of protections involved excluding harvesting from riparian zones, old growth forest, rainforest, including over ridge corridors to connect adjacent catchments^1^. A second tier of protection involved habitat retention at individual tree, clump and stand scales by targeting key habitat for a variety of threatened species that are expected to occur in a compartment. Under new Approvals (since 2018), a koala habitat model is used to identify potential habitat and trigger retention of important browse trees for the species at a rate of 10 per ha in the net harvest area (in addition to basal area limits for selective harvesting as described above) for high suitability habitat and 5 per ha in moderate suitability habitat. Previously harvested sites comprised a mosaic of regenerating eucalypts about 8 m tall, scattered seed trees and mature forest in exclusion zones. Environmental protections at that time were broadly similar, but koala scat surveys rather than a habitat model were required to trigger retention of small patches of ‘high use areas’ for the species, though only two such areas were triggered (at Cairncross) and larger areas of intensive harvest were permissible compared to that allowed under current regulations. All timber harvesting was undertaken under Integrated Forestry Operations Approval conditions as approved by NSW Environment Protection Authority. Research was undertaken with a NSW Department of Planning, Industry and Environment Scientific License (SL 100623) that provides institutional approval for scientific research on plants and animals, but because koalas were surveyed using passive acoustics no animal ethics approval was required. No plant or seed specimens were collected as part of the research.

Each site typically deployed sensors in a 5 × 5 array, with 400 m spacing, though this was varied to align with harvest plans (Figs. 4, 5). The spacing was selected to allow for correlated detections between adjacent sensors as required by SC models, given koala movements and that under ideal conditions koala bellows are recorded from 100 to ~ 300 m^40,41^ (Law unpubl. data). A single acoustic sensor (Song Meter SM4, Wildlife Acoustics, Maynard, USA) was deployed at each plot. Precise positions of sensors were sometimes shifted a little (< 200 m) to more closely align with harvested patches. Sensors were deployed at each array for 2 weeks in spring, the breeding season for koalas and when males are most vocal^15,23,38,41^. Sensors were programmed to record from sunset until sunrise, the peak calling period of koalas^23,41^, with a sampling rate of 22,050 Hz, and resolution of 16 bits per sample. Each State Forest and National Park pair was sampled simultaneously before switching to a new pair.

### Automated analysis of koala bellows

Recordings were scanned by acoustic software using a koala recogniser^41,42^. In this study, a version 2 recogniser of koala bellows was used, which was developed using a deep learning procedure (Himawan et al. unpubl. data). A recogniser developed for AviaNZ software was used to scan data post-harvest (L. Gonsalves unpubl. data). We made this change because AviaNZ processed our acoustic data much faster. We considered this would have no impact on the results for the following reasons: (1) detection rates for bellows were only slightly reduced with the AviaNZ recogniser, (2) spatial count modelling accounts for detection probability (see below) and (3) our design included spatial controls where data were analysed using identical methods as harvested sites, thus ensuring the comparisons were not confounded. Recordings matched by the koala recogniser were validated by manually visualizing spectrograms of the audio and listening to recordings to check for false positives. A single koala bellow often comprised multiple event triggers. Due to the large volume of events (true positives and false positives) that matched the koala recogniser, we validated seven nights where heavy rain and wind were absent from each study area. We considered a minimum independent time interval as 10 min between successive calls^31^ and considered those as independent events. That is, we validated recogniser hits until the first koala call was detected in each 10 min period. The number of 10 min periods per night with a koala call is highly correlated with total number of calls per night, so it can be used as an index of calling activity (authors unpubl. data). For SC analyses, we considered each night as a sampling occasion *K.*

### Spatial count model specifications

SC models use spatial correlation in temporally replicated counts (nights in our case) to estimate the number and location of the activity centres instead of individual identification of animals. Specifically, *N* is estimated as a subset of the data augmentation variable *M*, an oversized population of which our population is a part^43^. Abundance is estimated by summing inferred activity (i.e., home range) centers and density (*D*) is calculated by dividing *N* by the estimated study area, or state-space *S*, that encompasses potential activity centers for all individuals with a non-negligible probability of being detected by our detector traps over the study period.

In addition to estimating density, SC models, like all Spatial capture-recapture models, also estimate the baseline encounter rate—*λ*_0_, the probability of encounter of an individual if their home range centre is at the detector location—and a spatial scale parameter—*σ*, a measure of the rate of decay of encounter as the distance between the home range centre and the detector location increases^44^. The *σ* parameter is thus related to home range size and it is recommended that detectors are placed ~ 2*σ* apart^45,46^. We considered the detector locations, plus a 750 m buffer around the minimum rectangle envelope defined by the detector locations *J*, as the state-space *S* within which we estimated density (between 1100 and 1700 ha). We applied SC models using Poisson encounter models assuming bivariate normal movement in a Bayesian framework^19^.

We ran SC models using JAGS (ver 4.2.0^47^, interfacing through R using the *rjags* package^48^. We specified a λ_0_ prior with a uniform distribution between 0 and 100, a ψ prior with a beta distribution, shape and scale set to 1. We trialed four different *σ* priors: one uninformative with a uniform distribution between 0 and 100, and three with informative *σ* priors, following published methods^19,49^. The informative *σ* priors assumed a gamma distribution with the shape and spread varying based on home range size. We used a range of priors including uninformative, weakly informative (e.g. min–max male home ranges expected for the study area) and a strongly informative prior (site-specific mean male home range for north coast of 40 ha; Law et al. unpubl. data). We provide graphical results of estimated density for the strongly informative *σ* prior for all study areas. We set *M* = 500. We ran three chains of the JAGS models for 50,000 iterations with a burn in of 10,000 (after an adaptive phase of 1000) and did not thin the posterior distribution. Model convergence was assessed by visually inspecting trace plots for each monitored parameter, and we calculated the Gelman-Rubin statistic $\hat{R}$ using the *coda* package^48^, where values < 1.1 indicate convergence.

### Statistical analysis of BACIPS design

Testing for a change in male koala density followed the classic application of a BACIPS analysis that compares the Before and After differences using a two‐sample *t* test^50,51^. As a result, the difference between density at the site pairs (control-minus-harvested) at any one time is regarded as a replicate observation.

### Spatial variation in density

We overlayed the spatial variation in density at each array, as modeled by SC, with mapped forest age classes, environmental prescriptions (exclusions) and recent harvest polygons as recorded by GPS in harvesting machinery. The harvester polygons were not available for sites previously harvested 5–10 years earlier, so we instead used modelled disturbance layers (S. Hislop unpubl. data) extracted for the time of harvesting at these sites to delineate the most recent harvesting. Mean male koala density of each pixel in each forest category was then calculated before and after harvesting for each array. It should be noted that density values at a pixel scale are indicative of relative density within the study area and are most meaningful when averaged over larger contiguous blocks of a forest category rather than within small patches. Standard errors were derived for each category using the variation in density among polygons in each category.

## Data Availability

The datasets generated during and/or analysed during the current study are available from the corresponding author on reasonable request.
